# Endoscopic radial incision of a congenital antral web in a child

**DOI:** 10.1055/a-2253-0879

**Published:** 2024-02-22

**Authors:** Nuoya Zhou, Ou Chen, Xianglei Yuan, Bing Hu

**Affiliations:** 1 34753 Department of Gastroenterology and Hepatology, West China Hospital of Sichuan University, Chengdu, China; 2 590508 Department of Gastroenterology, Yaʼan Peopleʼs Hospital, Yaʼan, China

A 5-year-old boy presented with intermittent nonbilious vomiting for over 1 year. During the gastroscopy examination, an antral web with a central aperture was found with the pylorus visible in the distance ( Fig. 1 ). A barium swallow test showed narrowing in the area of antrum and pylorus, but the contrast agent could still pass through ( Fig. 2 ). The definitive diagnosis of “congenital antral web, incomplete antral web” was made. Endoscopic treatment was subsequently performed ( Fig. 3 , Video 1 ). The web was incised directly using an insulated-tipped knife (Olympus, Tokyo, Japan) in a radial pattern at eleven, three, and seven o'clock until the circular muscle was completely cut, clearly exposing the antrum and pylorus. The endoscope was able to pass the pylorus smoothly after the incision. The patient was discharged in stable condition after 6 days without any adverse events. The patient's symptoms were significantly resolved and a follow-up endoscopy at 6 months suggested good wound healing and widened aperture of the residual web ( Fig. 4 ).

**Fig. 1:**
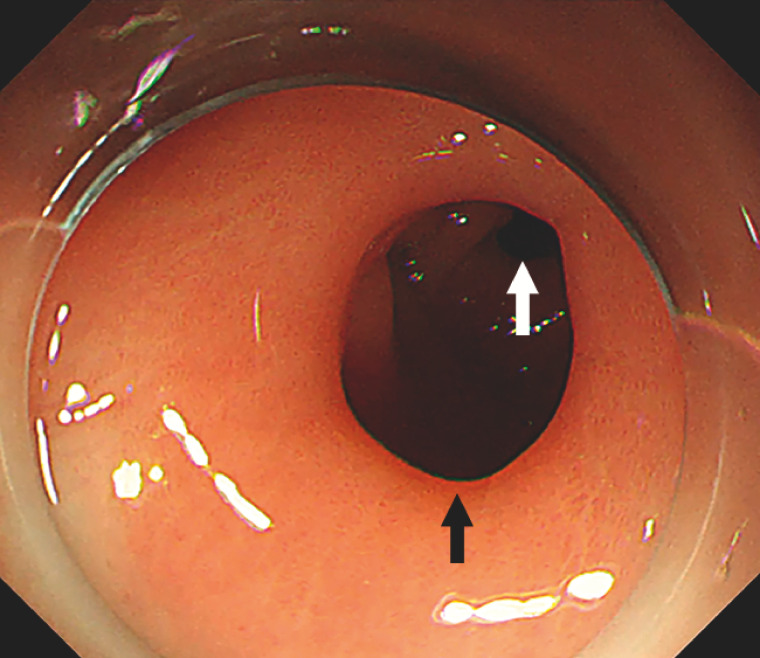
Endoscopic view of the antral web (black arrow) and the pylorus (white arrow).

**Fig. 2:**
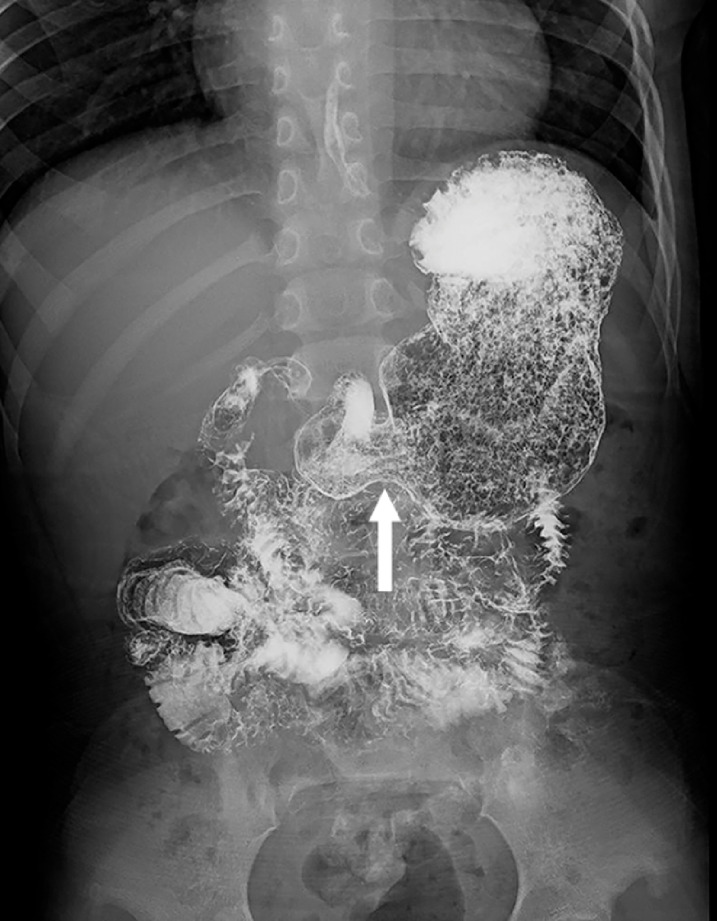
Barium swallow test showed narrowing in the area of antrum and pylorus (arrow).

**Fig. 3:**
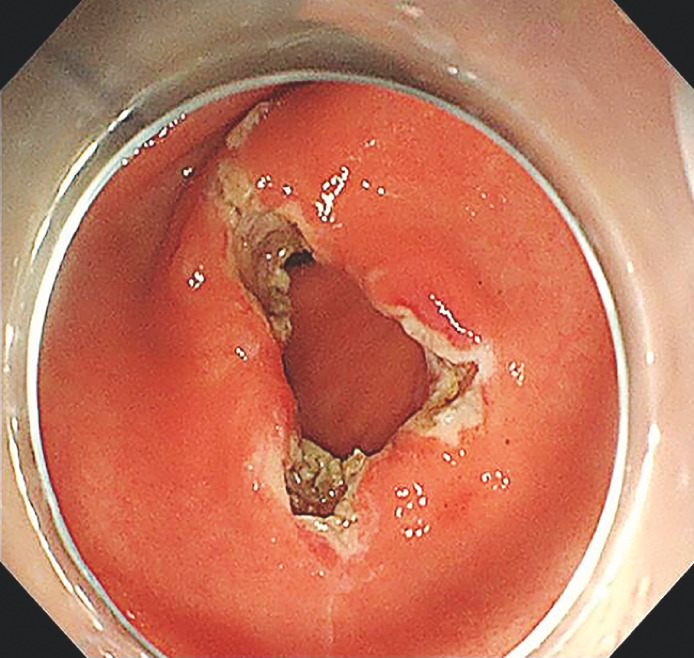
Endoscopic antral web radial incision.

Endoscopic radial incision of a congenital antral web in a child.Video 1

**Fig. 4:**
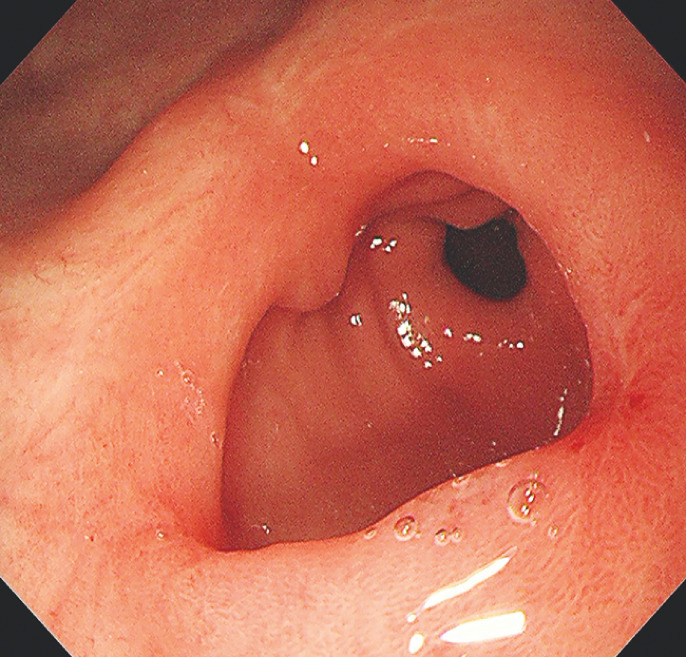
Endoscopic follow-up showed widened aperture of the residual web.

Antral web is a rare cause of gastric outlet obstruction. Its current treatment is mostly surgical web excision with pyloroplasty, but this method can be traumatic and often causes complications like bile reflux. Endoscopic treatment offers advantages of minimal damage to surrounding tissues and normal anatomy, shorter operation time, and faster postoperative recovery. There are few reports on endoscopic intervention for congenital antral web, usually combined with balloon dilation 1 . However, balloon dilation may have to be performed multiple times owing to unsatisfactory results. Our patient achieved successful results with a simple endoscopic radial incision, performed only once, with no signs of further narrowing. Endoscopic radial incision may be a safe and effective method for treating incomplete congenital antral web.

Endoscopy_UCTN_Code_TTT_1AO_2AH

## Notes

Endoscopy E-Videos https://eref.thieme.de/e-videos
*E-Videos* is an open access online section of the journal *Endoscopy* , reporting on interesting cases and new techniques in gastroenterological endoscopy. All papers include a high-quality video and are published with a Creative Commons CC-BY license. Endoscopy E-Videos qualify for HINARI discounts and waivers and eligibility is automatically checked during the submission process. We grant 100% waivers to articles whose corresponding authors are based in Group A countries and 50% waivers to those who are based in Group B countries as classified by Research4Life (see: https://www.research4life.org/access/eligibility/ ). This section has its own submission website at https://mc.manuscriptcentral.com/e-videos .
